# Gene cloning, expression and polyclonal antibody preparation of Rab3A for protein interaction analysis

**DOI:** 10.1186/s40064-016-3330-y

**Published:** 2016-10-03

**Authors:** Xia Tang, Jia Chen, Ying Wang, Xianchun Wang

**Affiliations:** Key Laboratory of Protein Chemistry and Developmental Biology of Ministry of Education, College of Life Sciences, Hunan Normal University, Changsha, 410081 Hunan China

**Keywords:** Rab3A, Fusion expression, Polyclonal antibody, Gel protein recovery, Protein interaction

## Abstract

**Background:**

Rab3A is a GTP-binding protein and plays critical roles in the regulation of synaptic vesicle exocytosis. Up to date, how Rab3A participates in such a regulatory process is not completely clear.

**Results:**

In this report the Rab3A gene from *Rattus norvegicus* was cloned and heterologously expressed in *E. coli* using pCold-TF expression vector with folding capacity. Due to the presence of His-tag sequence on the N-terminal side, Rab3A fusion protein was purified to greater than 95 % purity with a single Ni-affinity purification step. After the Rab3A fusion protein was used to immunize mice, an anti-serum against Rab3A with a titer of about 6000 was generated. Western blot analysis indicated that the prepared polyclonal antibody could recognize both Rab3A fusion protein and native Rab3A protein. To remove the tag sequence, thrombin was used to cleave the Rab3A fusion protein, followed by SDS-PAGE to separate the cleavage products. Using the gel protein recovery strategy with a Micro Protein PAGE Recovery Kit, the de-tagged Rab3A protein of electrophoretic purity was prepared.

**Conclusions:**

The present work not only prepared the ground for the study on Rab3A-mediated protein interactions, but also provided systematic experimental methods referable for the similar studies.

## Background

Rab proteins belong to the Ras superfamily of small GTPases and play central roles in the regulation of vesicle formation, actin- and tubulin-dependent movement and fusion (Stenmark and Olkkonen 2001). The major Rab protein in brain is Rab3A, which is able to associate with synaptic vesicle membranes by a C-terminal lipid modification (Geppert et al. 1994; Johnston et al. 1991). This protein associates with synaptic vesicles in its GTP-bound form and dissociates from the vesicles upon GTP hydrolysis or depolarization of the nerve terminal (Fischer von Mollard et al. 1994). Rab3A cycles between a soluble GDP-bound and a membrane-associated GTP-bound states, suggesting that there may be a mechanism that removes the protein from the membranes and thus neatly exerts its regulatory function (Geppert and Südhof 1998). Indeed, evidences show that the protein GDP dissociation inhibitor (GDI) inhibits GDP dissociation from Rab3A by binding to GDP-bound Rab proteins and removes them from membranes (Araki et al. 1990; Garrett et al. 1993). The GDP/GTP exchange protein (GEP) catalyzes the conversion of GDP-bound state to GTP-bound state, bringing Rab3A back to membranes (Wada et al. 1997), whereas GTPase activating protein (GAP) catalyzes the reverse conversion (Fukui et al. 1997). The three types of regulators, GDI, GEP and GAP, coordinately control the Rab3A cycling, whereas Rab3A interacts with multiple factors through the same effector domain (McKiernan et al. 1993).

How Rab3A participates in the regulation of synaptic vesicle exocytosis is not completely clear. It most likely acts via its effector proteins. Up to now several proteins have been found to be the effectors of Rab3A. Rabphilin-3A, as an upstream Rab3A effector, has an N-terminal Rab3A binding domain and strongly inhibits the Rab3A GTPase-activating protein (GAP)-stimulated GTPase activity of Rab3A (Kishida et al. 1993). RIM is referred to as a downstream Rab3A effector because it associates with Rab3A only at the active zone, regulating synaptic-vesicle fusion (Wang et al. 1997). Afterwards, Giovedì et al. (2004) confirmed that synapsin is a novel Rab3A effector protein on small synaptic vesicles. The presence of synapsin I stimulates GTP binding and GTPase activity of both purified and endogenous synaptic vesicle-associated Rab3A. In view of the crucial regulatory roles of Rab3A in exocytosis of synaptic vesicles, a great deal of genetic, overexpression, electrophysiological, and biochemical studies sought to address on the mechanism of action of Rab3A. However, the results are divergent and some are contradictory (Stenmark and Olkkonen 2001; Geppert and Südhof 1998; Bustos et al. 2014). Thus much work needs to be done in the relevant field.

There have been evidences suggesting that the critical regulation of the synaptic vesicle exocytosis is mediated by two proteins with opposite actions: synaptotagmin, a Ca^2+^-binding protein that is essential for Ca^2+^-triggered release and probably serves as the Ca^2+^-sensor in fusion, and Rab3, which limits the number of vesicles that can be fused as a function of Ca^2+^ in order to allow a temporally limited, repeatable signal. Thus, Rab3 and synaptotagmin are considered as the Yin and Yang of synaptic membrane fusion (Geppert and Südhof 1998). However, the related mechanism has not been completely understood. In the present research, we present the gene cloning, expression and polyclonal antibody preparation of Rab3A. The functionalities of the prepared products were evaluated in the interaction between Rab3A and synaptotagmin I, and results suggested that these products can be used as tools to further study the mechanism of synaptic vesicle exocytosis.

## Methods

### Materials and reagents

*E. coli* Top10 and *E. coli* BL21 (DE3) were purchased from Novagen, Inc. (San Diego, CA, USA) and stored in our lab. The expression vector pCold-TF was from Takara Bio (Dalian, China). Restriction enzymes *Nde*I and *Sal*I were from Thermo Fisher (MA, USA). Ni–NTA Agarose was from Thermo Fisher (Invitrogen, USA). Thrombin CleanCleave™ Kit was purchased from Sigma Aldrich (St Louis, MO, USA). Micro Protein PAGE Recovery Kit was from Sangon Biotech. Co. Ltd. (Shanghai, China). Other chemicals were of analytical or higher grade available.

### Cloning of Rab3A gene from *Rattus norvegicus*

*Rattus norvegicus* hippocampal tissues were used as Rab3A gene source. The total RNA was extracted from the hippocampal tissues and the first-strand DNA synthesis was performed according to the instructions of cDNA reverse transcription kit (Thermo Fisher Scientific, Waltham, MA, USA). The DNA was used as the template to obtain Rab3A gene by PCR amplification with the forward primer containing NdeI recognition site (5ʹ-GGGAATTCCATATGGCCTCAGCCACAGACTCTC-3ʹ) and the reverse primer containing SalI recognition site (5ʹ-ACGCGTCGACTCAGCAGGC GCAATCCTGAT-3ʹ). PCR was completed under the following conditions: preincubation for 3 min at 98 °C, followed by 32 cycles of 20 s at 98 °C, 20 s at 60 °C, 40 s at 68 °C, and then a final extension step of 72 °C for 30 min. The PCR products were subjected to electrophoresis on a 1.2 % agarose gel. The recovered and purified PCR fragment with a size of about 660 bp was ligated into pMD18T vector and then transformed into *E. coli* Top10, which were incubated in 1 ml LB fluid medium at 37 °C for 45 min with shaking (220 rpm) and then plated onto LB agar plates containing ampicillin (100 µg/ml). The single positive colonies were picked out and the plasmids were extracted. After digestion with *Nde*I and *Sal*I, the positive colonies were confirmed by DNA sequencing. Recombinant plasmid (pMD18T-Rab3A) was digested with restriction endonucleases NdeI and SalI and the resulting Rab3A gene fragments were ligated into the expression vector pCold-TF which had been digested with the same enzymes. Then the ligated plasmid pCold-TF-Rab3A was transformed into *E. coli* BL21(DE3).

### Expression and purification of Rab3A fusion protein

The recombinant plasmids encoding Rab3A were transformed into *E. coli* BL21 (DE3) by heat shock and the cells were plated onto LB agar plates with 100 µg/ml ampicillin, followed by incubation overnight at 37 °C. Single colonies from plates were transferred to 5 ml LB fluid medium containing 100 µg/ml ampicillin and incubated overnight at 37 °C with shaking at 220 rpm. This culture was diluted 1:100 into 500 ml LB broth plus ampicillin (100 µg/ml). The cells were grown at 37 °C until an OD_600_ of 0.6–1.0 was reached. After the temperature was reduced to 16 °C, the recombinant expression was induced by the addition of IPTG at a final concentration of 0.5 mM and the cells were grown overnight in an incubator with constant shaking of 220 rpm. The cells were harvested by centrifugation at 4000 g for 20 min at 4 °C. Cell pellet was re-suspended in a lysis buffer at a ratio of 5 ml buffer per 1 g cell pellet, followed by sonication. After centrifugation at 4000*g* at 4 °C, the supernatant was transferred into 15 ml Falcon tubes containing 200 µl of Ni–NTA Agarose and incubated on a rotating wheel for 3 h at 4 °C. The Ni–NTA Agarose with bound protein was separated from the lysate by centrifugation at 500*g* at 4 °C and washed three times. In the final step, the bound proteins were eluted from the Ni–NTA Agarose with 400 µl of 50 mM Hepes (pH 8.0) buffer containing 500 mM NaCl and 250 mM imidazole (elution buffer). An aliquot of the eluted proteins were analyzed by SDS-PAGE.

### SDS-PAGE and western blot analysis

Samples of Rab3A heterologous expression and purification were resolved on a 10 % SDS-PAGE gel in principle as described by Laemmli (1970), followed by visualization with Coomassie brilliant blue staining and scanning with a G:BOX Gel imaging system (Syngene, Cambridge, UK). For further identification of the expressed Rab3A fusion protein, the protein in the corresponding band was transferred from gel lane onto a nitrocellulose membrane (PALL Corporation, USA) using a blot electrotransfer apparatus in the wet transfer method (100 mA/2.5 h) and blocked in 5 % milk/TBST (50 mM Tris–HCl, 150 mM NaCl, 0.1 % Tween-20, pH 7.5) for 1.5 h at room temperature, and then probed with the mouse anti-His tag antibody (Novex, Life Technology, USA) (1:5000 dilution in 5 % milk/TBST) for 1.5 h at room temperature. After the membrane was washed three times (each for 6 min) using TBST, it was incubated with goat anti-mouse IgG conjugated with horseradish peroxidase (Promega Corporation, Madison, WI, USA) (1:8000 dilution in 5 % milk/TBST), followed by washing with TBST extensively. The blot was developed using the enhanced chemiluminescence (ECL) method (Thermo Scientific, USA) and recorded with a ChemiDoc XRS imaging system (Bio-Rad, USA).

### Antibody generation of Rab3A fusion protein and western blot analysis

The commonly used immunization protocols were employed to generate polyclonal antibody to Rab3A protein (Cooper and Paterson 2001). Briefly, the recombinant Rab3A (about 800 µg/ml in PBS) was mixed with equal volume of Freund’s incomplete adjuvant and injected subcutaneously into two 4- to 6-month-old mice. Initial immunization was followed by three booster injections at 2-week intervals. Antiserum was collected from the animals after 14 weeks. Antibody verification using western blotting was performed similarly to the identification of the expressed Rab3A fusion protein described as above, with the newly generated polyclonal antibody as the first antibody instead of anti-His tag antibody (1:4000 dilution in 5 % milk/TBST). Both recombinant and native Rab3A proteins were used as antigens.

### Tag sequence removal and de-tagged Rab3A protein purification

In order to remove the tag sequence from Rab3A fusion protein, the purified fusion protein was cleaved with thrombin. For optimizing the experimental conditions, the enzymolysis was performed and compared at different thrombin concentrations and for different times. After enzymolysis, the resulting protein mixture were electrophoresed on a 12 % SDS-PAGE, followed by gel protein recovery using a Micro Protein PAGE Recovery Kit according to the manufacturer’s instructions.

### GST pull-down and western blotting

GST pull-down experiment was used to detect a representative Rab3A-mediated protein interaction. For this detection, GST-Syt I-C2AB (the cytoplasmic C2 domains of synaptotagmin I) preparation and GST pull-down assay were performed according to the procedures previously described (Chapman and Jahn 1994). Glutathione-Sepharose bead-bound Syt I-C2AB was incubated with recombinant Rab3A protein overnight at 4 °C in a HNa buffer (10 mM Hepes–NaOH, 150 mM NaCl, 1 µM pepstatin A, 2 µM leupeptin, 0.3 mM phenylmethylsulfonyl fluoride, pH 7.4) containing 0.5 % Triton X-100. After incubation, the Glutathione-Sepharose beads were washed three times each with 1 ml of the HNa buffer containing 0.1 % TritonX-100. The proteins that remained bound to the beads were eluted with 2× SDS loading buffer and were analyzed by 10 % SDS-PAGE, followed by immunoblotting with the anti-Rab3A polyclonal antibody we prepared and anti-GST antibody (Abcam, England) as the primary antibodies, and horseradish peroxidase (HRP)-conjugated anti-mouse lgG antibody as the second antibody.

## Results

### Cloning of Rab3A gene

**Fig. 1:**
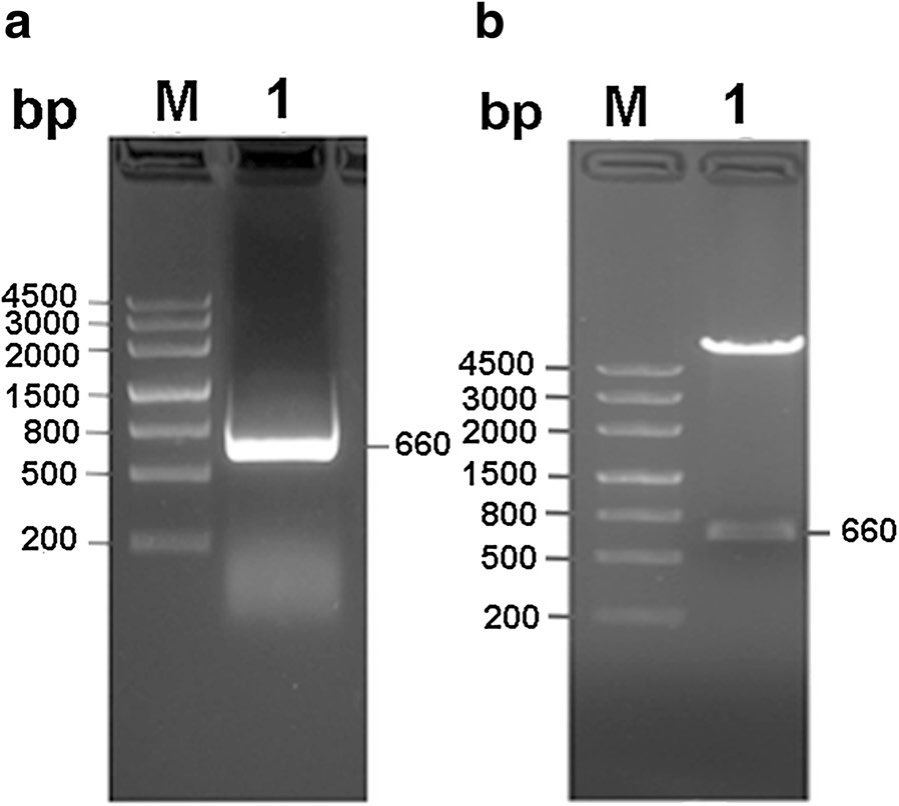
Agarose gel electropherograms of the products of Rab3A PCR amplification (**a**) and the recombinant vector digestion by *Nde*I and *Sal*I (**b**). *Lanes* labelled with 1 in (**a**) and (**b**) represent the products of PCR amplification and double digestion, respectively. M, DNA molecular weight marker

The cDNA for the hippocampal RNA was prepared using reverse transcription and used as the template to obtain Rab3A gene by PCR amplification using Rab3A-specific primers. Figure 1a shows the agarose gel electropherogram of PCR amplification products in which a bright band of about 660 bp was the band of interest. The resulting fragments encoding Rab3A were subcloned into pMD18T vector. The recombinant plasmid was identified by restriction enzyme digestion and sequencing. The double enzyme digestion of the recombinant plasmids demonstrated that the fragments were successfully ligated into the vectors (Fig. 1b). Sequencing confirmed that the sequence of cloned fragment was correct (data not shown). Then the gene was successfully cloned into the expression vector pCold-TF and transformed into *E. coli* BL21(DE3) for heterologous expression.

### Heterologous expression and affinity purification of Rab3A fusion protein

**Fig. 2:**
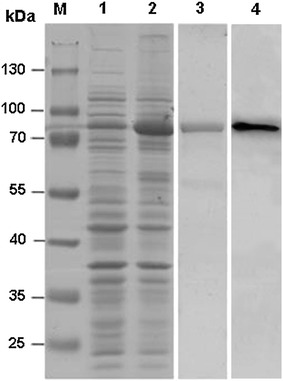
Induction expression, affinity purification and confirmation of Rab3A fusion protein. *Lane 1*, total proteins (35 μg) before induction; *lane 2*, total proteins (35 μg) after induction with IPTG; *lane 3*, the purified Rab3A fusion protein; *lane 4*, western blotting of Rab3A fusion protein. *M*, protein molecular weight marker

For optimizing the experimental conditions for heterologous expression, after the expression vector pCold-TF was transformed into *E. coli* BL21(DE3), the expression was induced with 0.5 mM IPTG (final concentration) at different combinations of temperature and time (37 °C for 4 h, 28 °C for 7 h and 16 °C overnight). The results showed that the expression efficiencies of Rab3A fusion protein were similar at above different combinations (data not shown). 16 °C-overnight (16 h) combination was chosen as the experimental conditions. The expressed fusion protein was purified using nickel-affinity Agarose beads, eluted with imidazole. The purity of the protein samples from each step was analyzed by SDS-PAGE (Fig. 2). As it is shown in Fig. 2, the content of Rab3A fusion protein in the sample after IPTG induction (lane 2) was much higher than that before induction (lane 1), suggesting the high expression efficiency. After a single-step affinity purification based on the His tag of Rab3A fusion protein, the purity of the protein sample was estimated to be greater than 95 % (lane 3). For further confirmation of the Rab3A fusion protein, we made a western blot analysis of the protein using anti-His tag antibody as the primary antibody. The result indicated that there was a band appearing in western blot at the position corresponding to that of the Rab3A fusion protein on the gel lane, suggesting that His-tagged Rab3A fusion protein was successfully expressed (lane 4).

### Preparation and characterization of the antibody against Rab3A protein

**Fig. 3:**
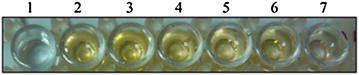
Titer determination of antiserum against Rab3A fusion protein. *1,* negative control; *2–7*, 1:1000, 1:2000,1:3000, 1:4000, 1:5000 and 1:6000, respectively

**Fig. 4:**
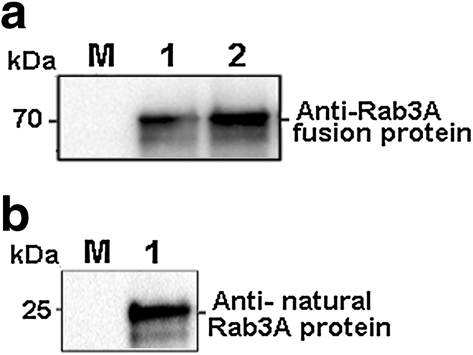
Western blot detection of Rab3A fusion protein (**a**) and native Rab3A protein (**b**) with prepared polyclonal antibody. *Lanes 1* and *2* in (**a**) were Rab3A fusion protein with different loading amounts

Using the purified Rab3A fusion protein as antigen to immunize mice, the antiserum against Rab3A protein was successfully prepared. Indirect ELISA assay demonstrated that the titer of the antiserum was about 1:6000 (Fig. 3). In order to detect the specificity of the prepared polyclonal antibody, we used the mouse serum as the primary antibody to detect the Rab3A fusion protein as well as the native Rab3A in the extract from rat hippocampal tissues in a western blot experiment. The results (Fig. 4) showed that the antibody recognizes both Rab3A fusion protein and native Rab3A protein, demonstrating that the newly prepared polyclonal antibody can be used in the research on the Rab3A-mediated protein interactions.

### Tag sequence removal and de-tagged Rab3A protein purification

**Fig. 5:**
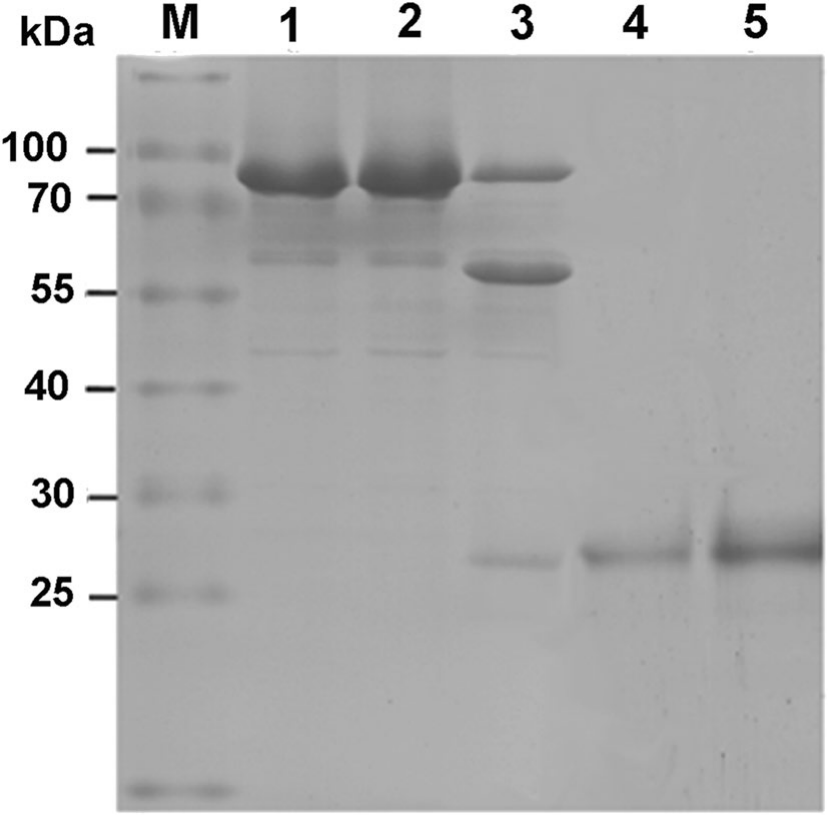
SDS-PAGE of Rab3A fusion protein before and after cleavage with thrombin. *Lanes 1* and *2*, Rab3A fusion protein with different loading amounts before cleavage (10 μg per lane). *Lane 3*, protein mixture after cleavage with thrombin (10 μg per lane). *Lanes 4* and *5*, purified de-tagged Rab3A protein with different loading amounts

The Rab3A fusion protein expressed with expression vector pCold TF has a fairly large tag sequence consisting of trigger factor (TF) and His_6_-tag, etc. For obtaining desired de-tagged Rab3A protein, thrombin was used to cleave the fusion protein according to the instructions of the enzyme supplier, followed by SDS-PAGE and gel protein recovery using a Micro Protein PAGE Recovery Kit to prepare the desired de-tagged Rab3A protein. SDS-PAGE analysis (Fig. 5) showed that, under the present experimental conditions, thrombin cleavage resulted in three main protein bands. Based on their molecular weights, it could be speculated that they were uncleaved fusion protein, tag sequence and de-tagged Rab3A protein, respectively (lane 3 in Fig. 5). After the gel slice containing de-tagged Rab3A was excised, destained and mashed, the de-tagged Rab3A protein with high purity was obtained from the gel by repeated extraction (lanes 4 and 5 in Fig. 5).

### Detection of Rab3A interaction with synaptotagmin I

**Fig. 6:**
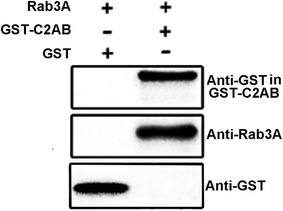
Detection of Rab3A interaction with the C2 domains of synaptotagmin I

In view of the fact that the C2 domains of the synaptotagmin I are its functional domains (Geppert and Südhof 1998; Chapman 2008), we used the C2 domains GST fusion protein (GST-Syt I-C2AB) to further evaluate the prepared polyclonal antibody and the functionality of recombinant Rab3A. After GST pull-down using GST-Syt I-C2AB as the bait protein and recombinant Rab3A as the prey protein, western blot analysis with the prepared polyclonal antibody as the first antibody showed that Rab3A could interact with the C2 domains (Fig. 6), demonstrating the functionality of purified Rab3A.

## Discussion

Due to the multiple important physiological roles of Rab3A protein in the cells, much attention has been attracted to the structure and functions of the protein. However, the low abundance of the protein in tissues that leads to the impossibility to obtain satisfactory amounts of the protein from the natural sources limits its deep research. Obviously, heterologous expression is a useful alternative to the native extraction (Paukner et al. 2015; Papaneophytou and Kontopidis 2014; Zhang et al. 2014) and provides a simple and inexpensive means to produce large amounts recombinant Rab3A protein.

Generally speaking, owing to the continuous development and improvement of PCR technique, the cloning of a gene with known nucleotide sequence is relatively easy. Comparatively, protein expression and purification are more difficult because they involve many issues, such as the selection of optimal expression organism, high efficiency expression vector, most appropriate growth condition, efficient purification strategy, etc., which in turn depend on the protein characteristics and downstream requirements (Ferrer-Miralles et al. 2015). The production of soluble and functional recombinant proteins is among the goals in the heterologous expression field. In the present work, we used a fusion expression vector (pCold TF) newly developed by Takara Bio Inc. (Shiga, Japan). pCold TF is a fusion cold shock expression vector that expresses trigger factor chaperone as a soluble fusion tag. This vector provides cold shock technology for high yield protein expression, combined with TF to facilitate correct protein folding (Saini et al. 2014; Qing et al. 2004). Our experimental results showed that Rab3A fusion protein was indeed efficiently expressed using pCold TF vector (Fig. 2). Furthermore, Rab3A fusion protein was efficiently purified in a single Ni-affinity purification step. Nevertheless, when we used thrombin to cleave the fusion protein, we found that the enzyme could not completely cleave the fusion protein even at higher concentrations. However, the data that Takara provided indicated that thrombin could almost completely remove the tag sequence of enzyme protein C fusion protein (Bio View Takara Bio Europe Edition), suggesting that the experimental conditions for the tag sequence removal of TF fusion proteins are dependent on the properties of the fusion proteins. Besides, Factor Xa was also tentatively used to cleave the fusion protein. However, the effect was found to be worse than that using thrombin (data not shown). Furthermore, when we tried to use Ni-affinity purification to separate the cleaved tag sequence as well as uncleaved fusion protein from the de-tagged Rab3A, we found that the purification efficiency was very limited, as it was hardly to obtain pure de-tagged Rab3A from the protein mixture, although we had made much effort on the optimization of experimental conditions (data not shown). It was speculated that the changes in the conformation of the tag sequences impeded their His tag binding to the agarose beads. In addition, gel filtration and RP-HPLC (using C4 column) were also tentatively employed to purify the de-tagged Rab3A protein, and, however, they all failed. Finally, the gel protein recovery strategy using a Micro Protein PAGE Recovery Kit was employed to recover the de-tagged Rab3A protein after SDS-PAGE. Using this method, the pure Rab3A protein can be easily obtained, although it is usually difficult to prepare large amounts of the sample. Fortunately, due to the development of the techniques in protein chemistry, many analyses based on micro amounts of sample have become possible. It is worth mentioning that it is better not to use reducing reagents such as DTT to treat protein sample before SDS-PAGE and not to fix the gel after SDS-PAGE, which is in favor of renaturation and extraction of the protein of interest. Furthermore, preparative gel electrophoresis can be used to enlarge the scale of sample preparation, and Native-PAGE would be helpful to produce Rab3A of higher quality.

Protein–protein interactions represent the key events in various cellular processes and the study on such interactions is of growing interest. Antibodies are often used to detect the interacting proteins in many protein interaction researches such as co-immunoprecipitation (Co-IP), pull-down and western blotting, etc. Although there are a wide variety of antibodies commercially available, some special antibodies and those needed in a relatively large amounts are usually made by oneself. Of the several kinds of methods for antibody preparation, polyclonal antibody preparation is often the first choice in a laboratory due to its easiness to operate, low cost and needing no complex devices (Cooper and Paterson, 2001; Novo et al. 2010). In our present work, in view of the high-level expression of the Rab3A fusion protein, we used the fusion protein rather than de-tagged Rab3A protein as antigen to raise polyclonal antibody. The titer determination and western blot detection indicated that the polyclonal antibody was successfully produced. More importantly, the antibody could specifically recognize both recombinant Rab3A in the fusion protein and native Rab3A protein, which suggested that the newly prepared polyclonal antibody can be used in the researches on the Rab3A-mediated protein–protein interactions.

## Conclusions

Rab3A gene from *R. norvegicus* was successfully cloned and highly efficiently expressed as a fusion protein in *E. coli* using cold shock expression vector pCold TF with folding capacity. The polyclonal antibody raised against the fusion protein could specifically recognize both Rab3A fusion protein and native Rab3A protein. Comparatively, thrombin is more efficient than Factor Xa in the cleavage of the tag sequence of the fusion. The de-tagged Rab3A protein can be completely purified from the cleavage product mixture using a combined strategy of SDS-PAGE separation and gel protein recovery. This work not only prepared a basis for the further research on Rab3A-mediated protein interactions, but also provides systematic experimental methods referable for the similar studies.

## Abbreviations

SDS-PAGE: sodium dodecyl sulfate polyacrylamide gel electrophoresis
GST: glutathione *S*-transferase
IPTG: isopropyl β-d-1-thiogalactopyranoside
ELISA: enzyme linked immunosorbent assay
Syt I: synaptotagmin I
Syt I-C2AB: cytoplasmic C2 domains of synaptotagmin I
